# Solution combustion synthesis of a nanometer-scale Co_3_O_4_ anode material for Li-ion batteries

**DOI:** 10.3762/bjnano.12.34

**Published:** 2021-05-10

**Authors:** Monika Michalska, Huajun Xu, Qingmin Shan, Shiqiang Zhang, Yohan Dall'Agnese, Yu Gao, Amrita Jain, Marcin Krajewski

**Affiliations:** 1 Department of Chemistry, Faculty of Materials Science and Technology, VŠB-Technical University of Ostrava, 17. listopadu 2172/15, 708 00 Ostrava-Poruba, Czech Republic; 2 Łukasiewicz Research Network ‒ Institute of Microelectronics and Photonics, Al. Lotników 32/46, 02-668 Warsaw, Poland; 3 Key Laboratory of Physics and Technology for Advanced Batteries (Ministry of Education), College of Physics, Jilin University, Changchun 130012, PR China; 4 Institute for Materials Discovery, University College London, London WC1E 7JE, United Kingdom; 5 Institute of Fundamental Technological Research, Polish Academy of Sciences, Pawińskiego 5B, 02-106 Warsaw, Poland

**Keywords:** anode material, cobalt oxide, lithium-ion battery, solution combustion synthesis, transition metal oxide

## Abstract

A novel solution combustion synthesis of nanoscale spinel-structured Co_3_O_4_ powder was proposed in this work. The obtained material was composed of loosely arranged nanoparticles whose average diameter was about 36 nm. The as-prepared cobalt oxide powder was also tested as the anode material for Li-ion batteries and revealed specific capacities of 1060 and 533 mAh·g^−1^ after 100 cycles at charge–discharge current densities of 100 and 500 mA·g^−1^, respectively. Moreover, electrochemical measurements indicate that even though the synthesized nanomaterial possesses a low active surface area, it exhibits a relatively high specific capacity measured at 100 mA·g^−1^ after 100 cycles and a quite good rate capability at current densities between 50 and 5000 mA·g^−1^.

## Introduction

Recently, a considerable research effort regarding new anode materials has been made because the traditional carbonaceous anodes cannot meet the requirements of the next-generation lithium-ion batteries (LiBs) due to their low capacity, sensitivity to electrolyte, and limited capability [1–3]. As a result, plenty of materials with high capacity and rate capability, good recyclability, and long lifetime have been proposed as potential next-generation anodes. Among them, transition metal oxides (TMOs) have attracted particular attention because their capacities are significantly greater than those of carbonaceous electrode materials. Also, most of the TMOs are relatively inexpensive and easily accessible due to their high natural abundance [1–3].

Considering the thermodynamic and chemical stability, as well as electrochemical properties, one of the important members of TMOs is cobalt(II,III) oxide (Co_3_O_4_) [4]. This oxide belongs to the group of spinels whose general formula is MNO_4_, where M and N are cations with different sizes and oxidation states. The spinel structure consists of a matrix composed of oxide ions O^2−^ (32e, Wyckoff sites) with cubic close-packed structure, stabilized with cobalt ions (Co^2+^) in tetrahedral positions (8a, Wyckoff sites) and cobalt ions (Co^3+^) in octahedral positions (16d, Wyckoff sites). Crystalline Co_3_O_4_ exhibits the space group *Fd*3*m* (227) [5]. It also can reversibly store eight lithium ions according to the following conversion reaction:

[1] $${\text{Co}}_{3}{\text{O}}_{4}+8{\text{Li}}^{+}+8e^{-}\overset{\text{discharge}}{\underset{\underset{\text{charge}}{\leftarrow }}{\to }}\;3\text{Co}+4{\text{Li}}_{2}\text{O}$$

This redox reaction corresponds to a theoretical capacity of about 890 mAh·g^−1^ [1–4]. However, similarly to silicon and tin materials, the lithium storage reactions associated with TMO electrodes are accompanied with large volume changes during lithiation–delithiation processes [1–4,6], but their volume variations are less significant [1]. This may lead to electrode pulverization and subsequent detachment of active materials from the current collector. Besides that, the Co_3_O_4_ electrode material suffers from low ionic and electronic conductivity, which influences its relatively slow charge/discharge rate [2,4]. In order to overcome the aforementioned drawbacks, some strategies have been proposed. One of them is related to the formation of composite materials consisting of Co_3_O_4_ and different materials, including carbon-based materials, such as graphene [7–8], carbon nanotubes [9], carbon coatings [10], dictyophora indusiata-derived carbon [11], or other transition metal oxides [12]. This approach usually leads to a conductivity enhancement and sometimes mitigates the impact of volume changes. However, at the same time, it causes a decrease of the Co_3_O_4_ capacity. Another strategy is associated with synthesis procedures that allow one to produce nanometer-scale Co_3_O_4_ materials with various shapes and morphologies. It has been established that the electrochemical performance of Co_3_O_4_ materials is improved when they possess either small size or appropriate pore size distribution and morphologies, such as porous or hierarchical structures, or the combination of both these features [3–4]. So far, different syntheses have been proposed including sol–gel methods [4,6,13–15], sol–electrospinning techniques [16–19], hydrothermal and solvothermal syntheses [20–28], precipitation and co-precipitation [29–31], chemical thermal decomposition and pyrolysis [32–37], and other methods [38–41]. Co_3_O_4_ nanomaterials with various shapes were obtained, such as films [6,16], particles [13–14,32,38], spheres [15,20,28,36], fibers [18–19], wires [21,30,40], tubes [22,32], cages [23,33], flakes [24], sheets [25,37,41], and flowers [31].

Intentionally, we have not mentioned before another synthesis technique, which is known as solution combustion synthesis (SCS) or self-propagating high-temperature synthesis (SHS). In fact, this method is relatively inexpensive and effective for the production of various industrially useful materials [42–50]. The SCS process is based on strongly exothermic redox reactions in which oxidants, such as metal nitrates, carbonates, or sulfates, react with reducing organic agents, frequently called fuels, such as starch, urea, glycine, or glucose [42–43,45–50]. During the typical SCS process, the initial phase is carried out at low temperatures and is associated with the evaporation of water or solvent. This allows for the formation of a gel, which acts as the precursor in the main part of the self-propagation reaction. This step is carried out at elevated temperatures and leads to the decomposition of the fuel resulting in the formation of gaseous by-products, such as CO_2_, NO_2_, or NH_3_, and the generation of heat. On one hand, this initiates a foaming reaction, on the other hand, this prevents from grain growth and agglomeration processes [10,42–43,45–50]. Therefore, one of the advantages of this method is that the obtained materials are usually well-crystalline fine-grained powders with a low degree of agglomeration. Besides that, the SCS method is a one-step process that does not need additional post-reaction treatments, for instance, annealing or calcination.

The SCS method has been successfully used to produce spinel-structured Co_3_O_4_ nanomaterials [48–52]. Taking advantage of these reports, we decided to design a new SCS synthesis path in which we applied for the first time ᴅ-(+)-glucose as the reducing agent instead of the previously used urea [48–49,51] and citric acid [50,52]. ᴅ-(+)-glucose has been used as alternative fuel for the SCS process mainly for two reasons. The first one is that ᴅ-(+)-glucose is a naturally abundant form of glucose, which is formed during photosynthesis by plants and most algae [53]. The second reason is associated with the fact that a lot of dangerous gaseous by-products that contain nitrogen are usually released to the atmosphere during the decomposition of urea. The usage of ᴅ-(+)-glucose as fuel in the SCS allowed us to obtain a Co_3_O_4_ nanomaterial with relatively low surface area, which, surprisingly, revealed high rate capability and long cycle life as anode electrode material for Li-ion batteries. The obtained results are discussed in detail in the following sections of this work.

## Results and Discussion

**Figure 1:**
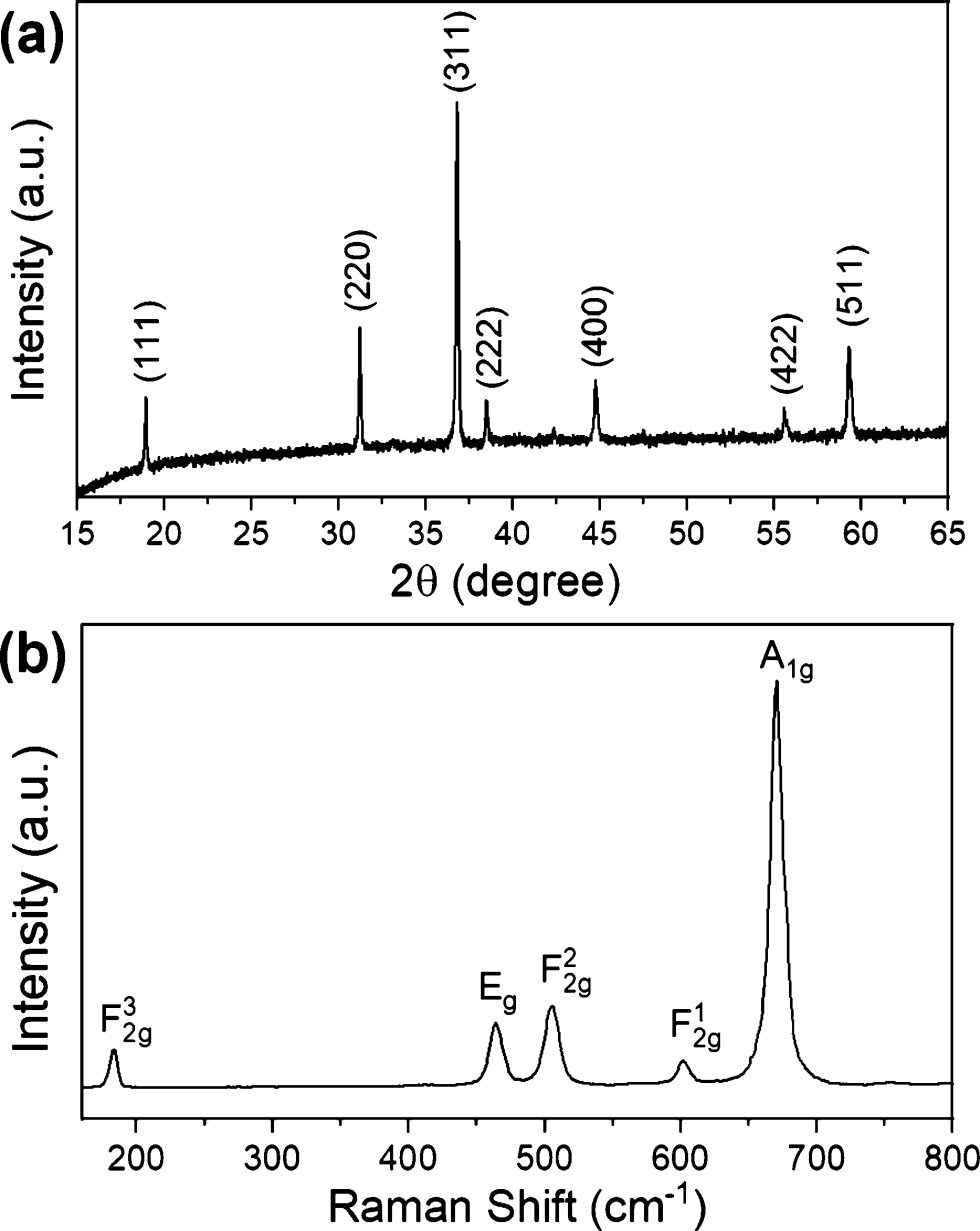
(a) XRD pattern and (b) Raman spectrum of Co_3_O_4_ powder.

The structure of as-prepared Co_3_O_4_ powder was verified by XRD and Raman spectroscopy (RS) measurements. The experimental results from both techniques are shown in Figure 1 and they are complementary. The XRD pattern presented in Figure 1a reveals seven peaks located at 19°, 31.3°, 36.8°, 38.7°, 44.8°, 55.8°, and 59.4°, which correspond to the (111), (220), (311), (222), (400), (422), and (511) crystal planes, respectively. Their positions are characteristic for the cubic spinel crystal structure with the space group *Fd*3*m*. The calculated unit cell parameter (*a* = 8.085 Å) is consistent with the standard value for cobalt(II,III) oxide (42-1467 ICDD). The analysis of peak broadening yielded an estimated average crystallite size of about 40 nm, according to the Scherrer formula. The Raman spectrum shown in Figure 1b exhibits five bands located at 184, 464, 506, 601, and 670 cm^−1^, corresponding to the F_2g_^3^, E_g_, F_2g_^2^, F_2g_^1^, and A_1g_ active vibrational modes, respectively [5,10,54–55]. The band at 670 cm^−1^ represents the characteristic symmetric Co–O stretching vibration of the CoO_6_ octahedra, whereas the band at 184 cm^−1^ is associated with the tetrahedral sites (CoO_4_). The other bands correspond to the mixed motions of oxygen at tetrahedral and octahedral sites [56]. It is also important to mention that no impurities have been detected using XRD and RS. This confirms the successful formation of the Co_3_O_4_ material which, in addition, is highly pure and well crystalline.

**Figure 2:**
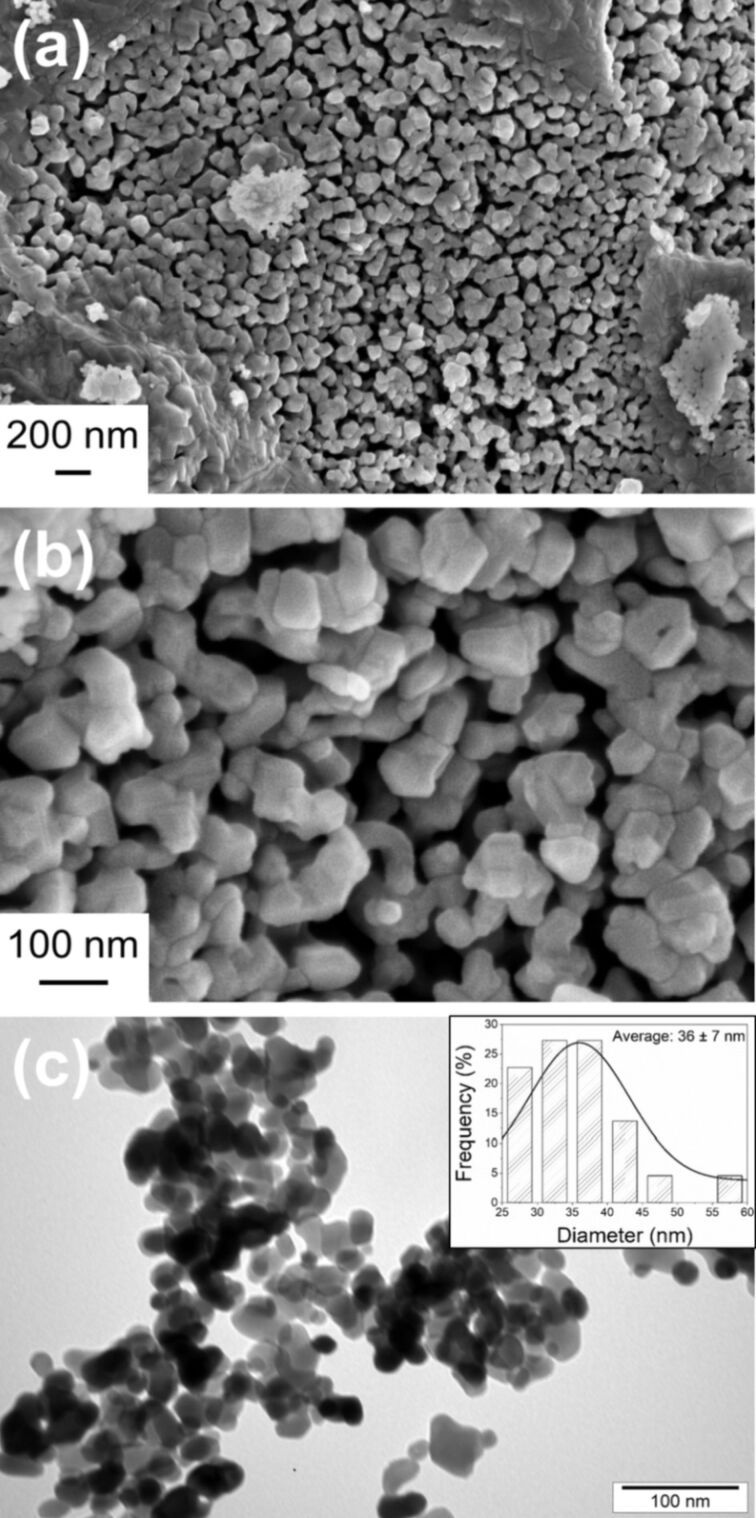
SEM images of Co_3_O_4_ powder magnified (a) 25,000 times and (b) 100,000 times. (c) TEM image of the Co_3_O_4_ powder (inset: a particle size distribution diagram).

The morphology of the investigated cobalt oxide has been determined with SEM and TEM (Figure 2). According to the recorded images, the Co_3_O_4_ powder is composed of nanoparticles ranging from 12 to 60 nm with an average diameter of about 36 nm. This size of nanoparticles is in a good agreement with the previously mentioned average crystallite size calculated with the Scherrer formula. It is also clearly seen that the particles are loosely arranged with a lot of free space between them. Interestingly, the specific surface area determined from BET measurements is about 3 m^2^·g^−1^, while the pore volume is found to be 0.02 cm^3^·g^−1^. These data are consistent with previously published works of Sahoo et al. [51] and Wen et al. [52]. Sahoo et al. [51] found that the size of Co_3_O_4_ materials synthesized through the SCS method increases, whereas its specific surface area decreases, with increasing process temperature. They reported that temperatures over 600 °C led to the formation of a material with a specific surface area of around 3 m^2^·g^−1^. Wen et al. [52] produced, through the SCS process, the porous Co_3_O_4_ nanoscale flakes with a specific surface area of 2.3 m^2^·g^−1^ and a pore volume of 0.02 cm^3^·g^−1^. Moreover, the TEM measurements indicate that the Co_3_O_4_ particles investigated in this work exhibit a compact structure rather than a porous one (Figure 2c).

**Figure 3:**
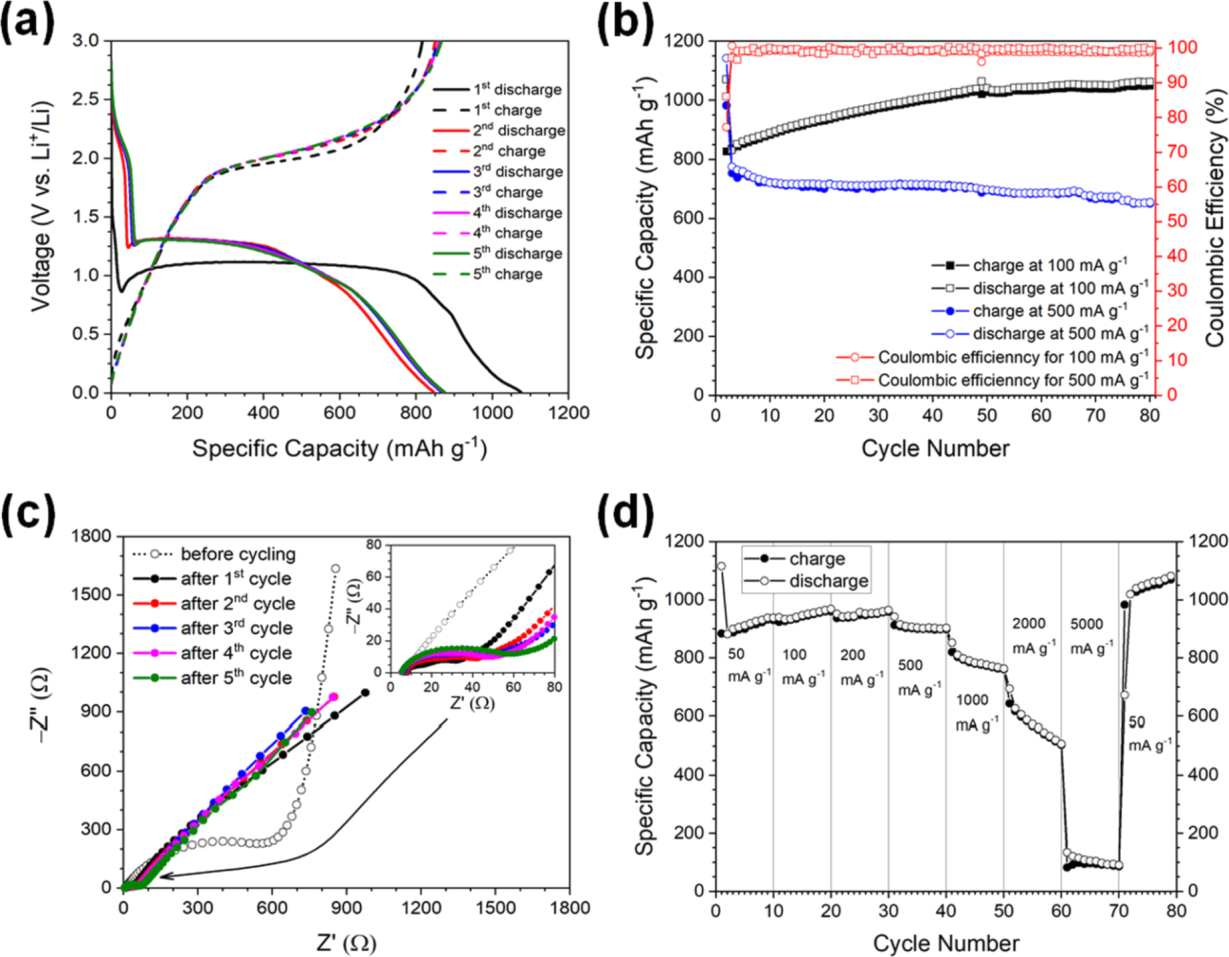
Electrochemical properties of the Co_3_O_4_ electrodes. (a) The first to fifth cycle profiles measured at current density of 100 mA·g^−1^. (b) Cycling performance measured at current density rates of 100 and 500 mA·g^−1^. (c) Electrochemical impedance spectra (Nyquist plots) of the cell with Co_3_O_4_ powder as electrode material measured before cycling and after the first, second, third, fourth, and fifth cycle. (d) Rate capability tests at different current density rates ranging from 50 to 5000 mA·g^−1^.

The electrochemical performance of the Co_3_O_4_ powder as anode material was evaluated in a two-electrode coin cell in a voltage window from 0.01 to 3.0 V vs Li^+^/Li. The first to fifth charge–discharge cycle profiles measured at a current density of 100 mA·g^−1^ are shown in Figure 3a. During the first discharge, the profile consists of one long plateau at around 1.0 V, corresponding to the formation of metallic cobalt and Li_2_O according to the reversible conversion reaction stated before (Equation 1), and a gradual continuous voltage slope down to 0.01 V, corresponding to electrolyte decomposition and the resulting formation of a solid–electrolyte interface (SEI) layer [14–15,19–21,26,28–31,34–41,52]. In turn, the following discharge profiles slightly differ from the initial one. They are much shorter and are shifted towards a higher voltage of about 1.28 V. This observation is not new and it is attributed to the changes of structure and/or composition of the electrode material that occur after the initial cycle [17,25,52]. They are also very similar to each other due to the stabilized reversible conversion and re-conversion reactions [14]. The initial discharge and charge capacities are about 1075 and 817 mAh·g^−1^, respectively. This corresponds to an initial Coulombic efficiency of about 76%. At this point, it is worth noting that the capacity loss of about 25% is very characteristic for the first cycle of Co_3_O_4_ electrodes and it has been frequently reported for Co_3_O_4_ nanostructures with various shapes [15,21,23–27,30–31,34,36]. This capacity fade is usually ascribed to an irreversible electrolyte decomposition, the formation of the SEI layer, and the formation of stable Li_2_O [4,14–15,19–21,26,28–31,34–41]. The charge profiles are very similar and they exhibit a voltage plateau at about 2.0 V, which is associated with the re-conversion reaction and the resulting reconstruction of the cobalt oxide electrode. As mentioned before, the shapes of the second and the following charge and discharge profiles are almost identical. This reveals a superior cycle stability of the investigated Co_3_O_4_ electrode during the lithiation/delithiation processes.

The results of cyclability tests of the Co_3_O_4_ electrode at current densities of 100 and 500 mA·g^−1^ measured up to 100 consecutive charge–discharge cycles are given in Figure 3b. At 500 mA·g^−1^, the measured values of the specific capacity are lower than the theoretical capacity of a Co_3_O_4_ electrode (890 mAh·g^−1^). In general, the specific capacity of the investigated material oscillates around 690 mAh·g^−1^ up to the 70th cycle. Then, it gradually fades and reaches about 533 mAh·g^−1^ after 100 cycles. Nevertheless, the measured capacity is still 1.43 times higher than the theoretical capacity of commercially used graphite electrodes (372 mAh·g^−1^ [10,21]). This behavior is frequently observed for pure and unmodified Co_3_O_4_ electrodes tested at current densities above 200 mA·g^−1^ [4,24,27]. It can be related to low ionic and electronic conductivity, which influences the charge/discharge at high current densities [2,4]. Interestingly, the cyclability test performed at 100 mA·g^−1^ shows that the values of specific capacity consecutively rise over the theoretical capacity value and are maintained at 1060 mAh·g^−1^ after 100th cycle. This phenomenon is well known for different transition metal oxide electrodes and is usually ascribed to the reversible formation/dissolution of a gel-like SEI layer, which provides some additional reversible capacity apart from the reversible conversion reactions occurring on the electrodes [4,15,17,19,21,24,26,29,34,37,40,52]. This additional lithium storage is often referred to as “pseudo capacitive” mechanism [25,29]. It is also worth noting that after the initial decrease of the Coulombic efficiency observed for the first cycle, it is maintained at nearly 100% of the new level in the following cycles. The measured capacity values are at a level similar to those that have been already reported for different Co_3_O_4_ powder materials (cf. Table S1 in Supporting Information File 1). Nevertheless, it is important to point out that the cobalt oxide nanostructure synthesized in this work has one of the lowest specific areas. This indicates a good reversibility of the lithium storage and release in the case of the investigated Co_3_O_4_ electrode.

In order to better understand of the capacitive behavior of the investigated Co_3_O_4_ electrode material, electrochemical impedance spectroscopy (EIS) measurements at a current density of 100 mA·g^−1^ were carried out before cycling and after the first, second, third, fourth, and fifth cycle. The obtained Nyquist plots are presented in Figure 3c. The spectrum measured before cycling consists of a semicircle in the high- and medium-frequency ranges followed by an inclined line at low frequencies. This plot can be described by the equivalent circuit shown in Figure S1a in Supporting Information File 1. These EIS features represent the charge transfer at the electrode–electrolyte interface (*R*_ct_) and the Warburg impedance (*W*), which is attributed to the diffusion of Li^+^ ions in the bulk electrode material [25,38]. The plots for the cycled cell are slightly different. They are composed of two overlapping semicircles measured at the medium and the high frequencies and of an oblique line in the low-frequency region (cf. the equivalent circuit shown in Figure S1b in Supporting Information File 1). Again, the semicircle at medium frequencies and the oblique line at the low frequencies represent the charge transfer at the electrode–electrolyte interface (*R*_ct_) and the Warburg impedance (*W*), respectively. The semicircle at high frequencies is mainly attributed to SEI resistance (*R*_SEI_) and contact resistance (*R*_f_) [13,15,20–21,25,38]. At this point, it should be also mentioned that the separation of two semicircles in the medium- and high-frequency ranges is clearly visible only for the plots measured after the first and the second cycle, in which the SEI layer starts to form. Moreover, it is important that, before cycling, the slope angle of the inclined line in the low-frequency range equals almost 90°. This corresponds to a capacitive behavior of the electrode material. After cycling, the slope angle changes to almost 45°, which is typically associated with a semi-infinite diffusion behavior [25]. Further analyses of the EIS results yield that the value of *R*_ct_ has drastically decreased for the cycled cell. This indicates that the charge transfer reaction has been improved due to cycling. Also, a slight cycle-to-cycle increase of *R*_ct_ as well as of *R*_SEI_ is observed for the cycled sample. This phenomenon might be explained by the growth of the SEI layer, which provides some additional reversible capacity (cf. Figure 3c and [25,29]) and, at the same time, causes a small increase of the impedance.

The high rate capability is an important limitation for high-power applications such as electric vehicles. Therefore, rate capability measurements have been performed for the studied Co_3_O_4_ electrode at different current densities ranging from 50 to 5000 mA·g^−1^. The obtained results are shown in Figure 3d. It is clearly seen that the charge as well as discharge capacities greatly decrease for current densities above 500 mA·g^−1^. In contrast, in the case of current densities equal or below 200 mA·g^−1^ it seems that the capacity values rise from cycle to cycle. Returning to higher current densities, it is also visible that the capacities slightly fade for the first few cycles and then stabilize at a certain level, namely 898 mAh·g^−1^ at 500 mA·g^−1^, 761 mAh·g^−1^ at 1 A·g^−1^, 505 mAh·g^−1^ at 2000 mA·g^−1^, and 90 mAh·g^−1^ at 5000 mA·g^−1^. When the rate rises up again to 50 mA·g^−1^ after the cycling at high current densities, the charge and discharge capacities increase above the values of the initial cycles carried out at the same rate and establish at a level of about 1070 mAh·g^−1^. The capacity values measured at different current densities are slightly higher than those measured for the electrode composed of Co_3_O_4_ nanoscale flakes, synthesized in the SCS process by Wen et al. [52]. Undoubtedly, this observation indicates that the Co_3_O_4_ anode investigated in this work exhibits excellent reversibility and rate capability. This can be associated with the specific arrangement of particles that provides effective electrolyte-accessible channels for ion transportation and shortens the distance for Li^+^ ion diffusion and conversion reaction. Moreover, this particular structure inhibits a volume expansion during cycling, which leads to an elongation of the Co_3_O_4_ electrode life.

## Conclusion

A novel inexpensive solution combustion synthesis yielding cobalt oxide (Co_3_O_4_) nanoscale powder was proposed in this work. The as-prepared material was characterized by several complementary experimental techniques. It consisted of loosely arranged nanoparticles with an average diameter of about 36 nm and a specific surface area of about 3 m^2^·g^−1^. The Co_3_O_4_ material was also examined as anode material for Li-ion batteries. The obtained electrochemical results indicated that even though the synthesized nanomaterial possessed a very low surface active area, in comparison with previously reported Co_3_O_4_ nanostructures tested as anode materials, it exhibited a relatively high specific capacity of 1060 mA·g^−1^ measured at 100 mA·g^−1^ after 100 cycles and a remarkably good cyclability tested at current densities between 50 and 5000 mA·g^−1^.

## Experimental

**Figure 4:**
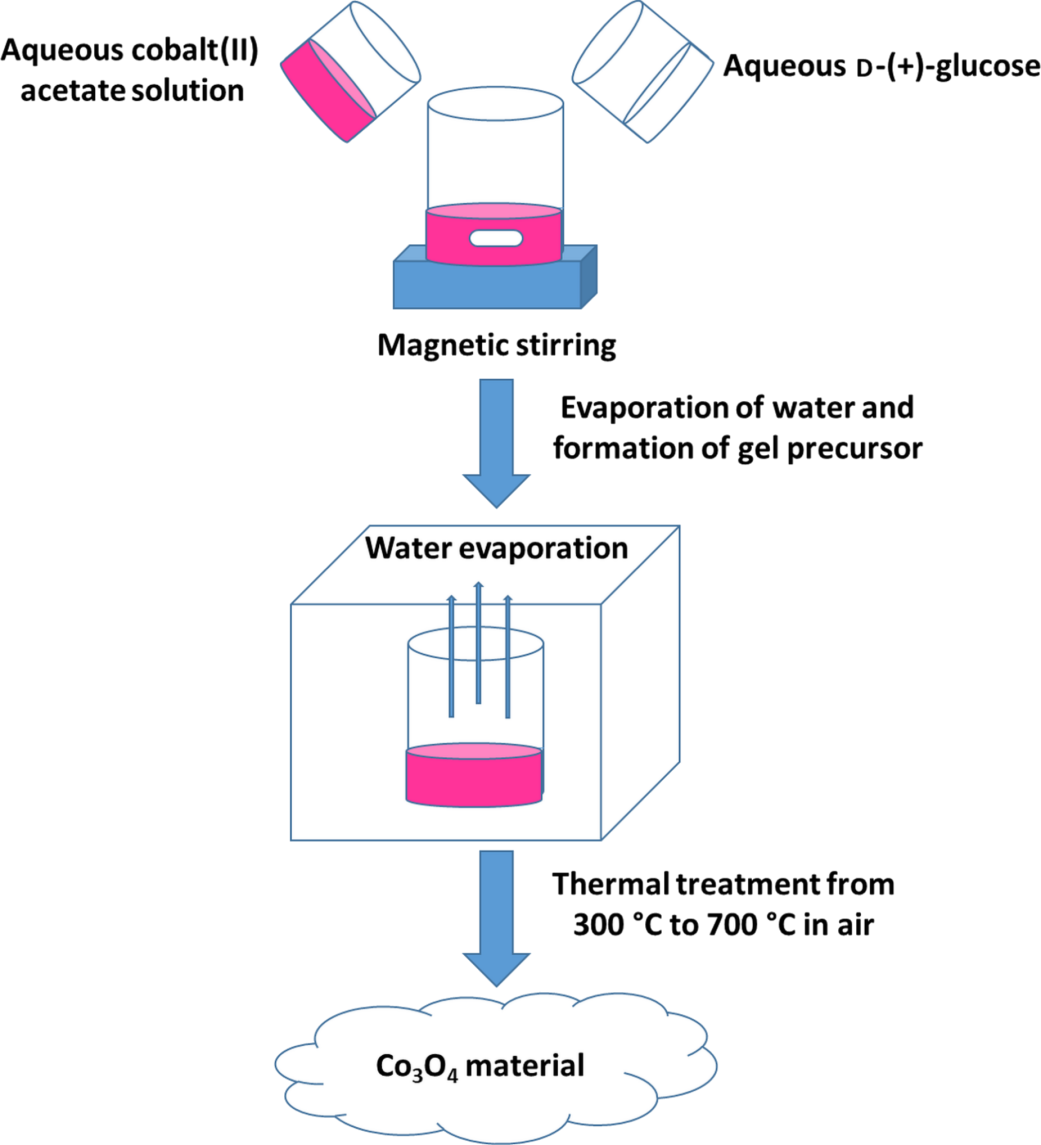
Flowchart of a solution combustion synthesis (SCS) of Co_3_O_4_ nanomaterial.

**Synthesis of the Co_3_O_4_ material:** High purity cobalt(II) acetate tetrahydrate (C_4_H_6_O_4_Co·4H_2_O, reagent grade) and ᴅ-(+)-glucose (C_6_H_12_O_6_; ≥99.5% (GC), Sigma-Aldrich) were used to synthesize the Co_3_O_4_ nanomaterial through a solution combustion method. In the process, 15.5 g of cobalt acetate (CoAc) and 10 g ᴅ-(+)-glucose (C) were separately dissolved in 40 and 20 mL of deionized water, respectively. Then, both solutions were mixed together. The molar ratio of Co/C was kept at 1:1. An evaporation process was applied to remove the water and to form a gel. After that, the resulting gel precursor was heated in an alumina crucible from 300 to 700 °C for 5 h in air. The flowchart of the synthesis is presented in Figure 4.

**Characterization of the Co_3_O_4_ material:** The structural properties of the Co_3_O_4_ material were determined using powder X-ray diffraction (XRD) and Raman spectroscopy (RS). XRD data was acquired using a SIEMENS D500 diffractometer equipped with a Cu Kα (λ_XRD_ = 1.542 Å) radiation source. The room-temperature XRD pattern in the range of 15° ≤ 2θ ≤ 60° was collected with a step size of 0.002° and an acquisition time of 3 s per step. The RS experiments were carried out using a confocal Raman spectrometer (Renishaw inVia) equipped with a charge-coupled device (CCD) camera and a continuous-wave diode-pumped Nd:YAG laser working at λ_RS_ = 532 nm. The Raman spectrum was collected at room temperature in air with acquisition time of 90 s, power of 0.5 mW, and a spot size of about 1 µm.

Surface morphology and particle size of the synthesized cobalt oxide powder were observed using a Carl Zeiss CrossBeam Auriga scanning electron microscope (SEM) operated at 5 kV and a JEOL – JEM 1011 transmission electron microscope (TEM) operated at 80 kV. The specific surface area of the investigated material was determined with the Brunauer–Emmett–Teller (BET) method based on N_2_ adsorption–desorption measurements performed with a Thermo Scientific Sorptomatic 1990 analyzer.

**Battery assembly and electrochemical measurements:** In order to use the Co_3_O_4_ sample as a working electrode, a slurry composed of previously prepared Co_3_O_4_ material, super P carbon (Saibo), and polyvinylidene difluoride (PVDF; Arkema) (weight ratio of 8:1:1) mixed in *N*-methyl-2-pyrrolidone was prepared and then coated on copper foil by using a doctor blade. After that, the electrode was dried at 120 °C in vacuum overnight. Disks with a diameter of 1 cm were cut and served as working electrodes in a two-electrode coin cell (CR2032). Metallic lithium (Sigma-Aldrich) simultaneously acted as counter and reference electrodes. Glassy fibers (Celgard) were used as a separator and soaked with 1.0 M LiPF_6_ dissolved in a mixture of ethylene carbonate (EC)/diethyl carbonate (DEC) (weight ratio of 1:1) (DoDoChem). The electrochemical cells were assembled in an argon-filled glove box.

Galvanostatic charge–discharge (GCD) tests were performed between 0.01 and 3 V at different current density rates using a Lanhe CT2001A. Electrochemical impedance spectroscopy (EIS) experiments were carried out to in the frequency range from 1 MHz to 1 mHz with a perturbation amplitude set at 10 mV on a potentiostat (VSP, Biologic-SAS).

## Supporting Information

**Table S1**: Comparison of the electrochemical performance of different Co3O4 powders applied as anode materials in Li-ion batteries (1 C = 890 mA·g^‒1^); **Figure S1:** Equivalent circuit corresponding to the electrochemical impedance spectroscopy (EIS) measurements for the cell (a) before cycling, and (b) after cycling; *R*_f_ – contact resistance, *R*_SEI_ – the solid–electrolyte interface (SEI) resistance, *C*_SEI_ – the surface capacitance, *R*_ct_ – the charge transfer resistance at the electrode–electrolyte interface, *C*_ct_ – the double layer capacitance of the electrode, *W* – Warburg impedance.

File 1 Additional data.
