# Supplementary material for: Solution combustion synthesis of a nanometer-scale Co3O4 anode material for Li-ion batteries
Source: Beilstein J Nanotechnol. 2021 May 10;12:424–31. doi: 10.3762/bjnano.12.34 (PMC8144916; doi:10.3762/bjnano.12.34)
Supplement: File 1 — Additional data. [file Beilstein_J_Nanotechnol-12-424-s001.pdf]

BEILSTEIN JOURNAL OF NANOTECHNOLOGY

Supporting Information

for

Solution combustion synthesis of a nanometer-scale Co_3O_4 anode material for Li-ion batteries

Monika Michalska, Huajun Xu, Qingmin Shan, Shiqiang Zhang, Yohan Dall'Agnese, Yu Gao, Amrita Jain and Marcin Krajewski

Beilstein J. Nanotechnol. **2021,** *12,* 424–431. doi:10.3762/bjnano.12.34

Additional data

License and Terms: This is a supporting information file under the terms of the Creative Commons Attribution License (https://creativecommons.org/licenses/by/4.0). Please note that the reuse, redistribution and reproduction in particular requires that the author(s) and source are credited and that individual graphics may be subject to special legal provisions.
The license is subject to the *Beilstein Journal of Nanotechnology* terms and conditions: (https://www.beilstein-journals.org/bjnano/terms)

Table S1: Comparison of the electrochemical performance of different Co_3O_4 powders applied as anode materials in Li-ion batteries (1 C = 890 mA·g^{-1}).

**Synthesis method**	**Powder shape**	**Charge-discharge rate (mA g^{-1})**	**Surface area (m^2 g^{-1})**	**Number of cycles**	**Specific capacity (mAh g^{-1})**	**Reference**
sol-gel	mesoporous octahedra	200	48.5	60	1195	[1]
sol-gel	agglomerated nanoparticle	178 (0.2 C)	–	over 120	620	[2]
sol-gel	bowl-like hollow microsphere	178 (0.2 C)	12.8	50	1441	[3]
electrospinning	microfiber	50	–	20	741	[4]
electrospinning	nanowire	222 (0.25 C)	68	60	805	[5]
electrospinning	nanotube	222 (0.25 C)	95	60	856	[5]
electrospinning	nanofiber	445 (0.5 C)	46.5	40	604	[6]
electrospinning	porous nanofiber	500	29.8	over 350	600	[7]
hydrothermal	microsphere	50	93.4	25	550	[8]
hydrothermal	nanowire array	500	–	100	1031	[9]
solution	nanocage	178 (0.2 C)	–	50	864	[10]
ammonia-assisted hydrothermal	snowflake-like nanosheet	500	22.5	100	1044	[11]
hydrothermal	mesoporous nanosheet	100	11.9	100	1067	[12]
hydrothermal	porous nanoplate	178 (0.2 C)	14.5	80	1001	[13]
hydrothermal	nanosphere	400	10.7	140	970	[14]
hydrothermal	nanocube	400	7	140	490	[14]
solvothermal	mesoporous hollow sphere	1500	23.9	90	1320	[15]
precipitation	nanoparticle	89 (0.1 C)	–	130	423	[16]
surfactant-assisted precipitation	nanowire	110	8	50	611	[17]
co-precipitation	flower-like nanoparticle	445 (0.5 C)	31.9	350	1227	[18]
alumina-temple	nanorod	50	–	100	815	[19]
alumina-temple	nanotube	50	–	100	850	[19]
alumina-temple	nanoparticle	50	–	100	830	[19]
thermal decomposition	nanocage	50	31.9	30	970	[20]
precipitation	nanooctahedra	100	–	200	956	[21]
ultrasonic spray pyrolysis	porous microsphere	400	43.3	50	654	[22]
spray drying	mesoporous nanosheet	3500	13	100	868	[23]
urea-assisted refluxing	hydrotalcite-like nanoparticle	100	22.4	100	741	[24]

Synthesis method	Powder shape	Charge-discharge rate (mA g^{-1})	Surface area (m^2 g^{-1})	Number of cycles	Specific capacity (mAh g^{-1})	Reference
microwave-assisted and urea-assisted hydrolysis	porous nanowire	400	41	100	600	[25]
surfactant-assisted self-assembly	nanosheet	100	94.3	30	1868.6	[26]
solution combustion	nanoflake	178	2.3	100	908	[27]
solution combustion	nanoparticle	100	3	100	1060	This work

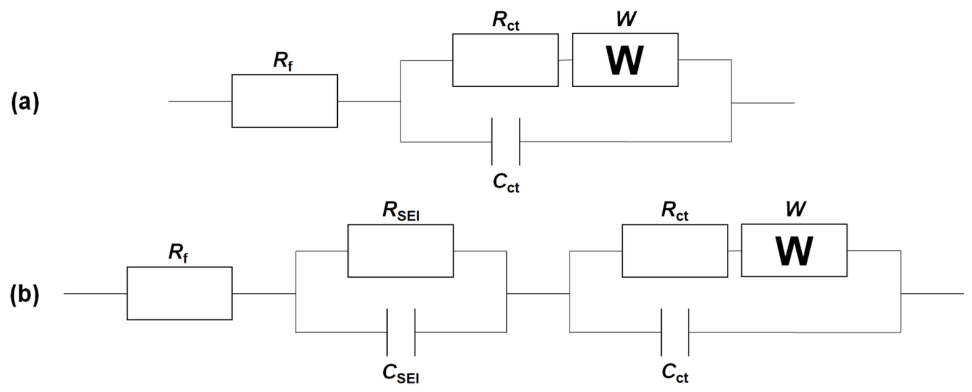

Figure S1: Equivalent circuit corresponding to the electrochemical impedance spectroscopy (EIS) measurements for the cell (a) before cycling, and (b) after cycling; R_f – contact resistance, R_{SEI} – the solid-electrolyte interface (SEI) resistance, C_{SEI} – the surface capacitance, R_{ct} – the charge transfer resistance at the electrode/electrolyte interface, C_{ct} – the double layer capacitance of the electrode, W – Warburg impedance.

References

1. Guo, J. X.; Chen, L.; Zhang, X.; Jiang, B.; Ma, L. Z. *Electrochim. Acta* **2014**, *129*, 410–415.
2. Devi, V. S.; Athika, M.; Duraisamy, E.; Prasath, A.; Sharma, A. S.; Elumalai, P. *J. Energy Storage* **2019**, *25*, 100815.

3. Wen, J. W.; Zhang, D. W.; Zang, Y.; Sun, X.; Cheng, B.; Ding, C. X.; Yu, Y.; Chen, C. H. *Electrochim. Acta* **2014**, *132*, 193–199.

4. Gu, Y. X.; Jian, F. F.; Wang, X. *Thin Solid Films* **2008**, *517*, 652–655.

5. Chen, M. H.; Xia, X. H.; Yin, J. H.; Chen, Q. G. *Electrochim. Acta* **2015**, *160*, 15–21.

6. Ding, Y. H.; Zhang, P.; Long, Z. L.; Jiang, Y.; Huang, J. N.; Yan, W. J.; Liu, G. *Mater. Lett.* **2008**, *62*, 3410–3412.

7. Fan, L.; Zhang, W. D.; Zhu, S. P.; Lu, Y. Y. *Ind. Eng. Chem. Res.* **2017**, *56*, 2046–2053.

8. Liu, Y.; Mi, C. H.; Su, L. H.; Zhang, X. G. *Electrochim. Acta* **2008**, *53*, 2507–2513.

9. Zhan, L.; Wang, S. Q.; Ding, L. X.; Li, Z.; Wang, H. H. *Electrochim. Acta* **2014**, *135*, 35–41.

10. Liu, D. Q.; Wang, X.; Wang, X. B.; Tian, W.; Bando, Y.; Goldberg, D. *Sci. Rep.* **2013**, *3*, 2543.

11. Wang, B.; Lu, X. Y.; Tang, Y. *J. Mater. Chem. A* **2015**, *3*, 9689–9699.

12. Wu, S. M.; Xia, T.; Wang, J. P.; Lu, F. F.; Xu, C. B.; Zhang, X. F.; Huo, L. H.; Zhao, H. *Appl. Surf. Sci.* **2017**, *406*, 46–55.

13. Liang, C. C.; Cheng, D. F.; Ding, S. J.; Zhao, P. F.; Zhao, M. S.; Song, X. P.; Wang, F. *J. Power Sources* **2014**, *251*, 351–356.

14. Zhang, B.; Zhang, Y. B.; Miao, Z. Z.; Wu, T. X.; Zhang, Z. D.; Yang, X. G. J*. Power Sources* **2014**, *248*, 289–295.

15. Shin, H.; Lee, W. J. *Mater. Chem. Phys.* **2018**, *214*, 165–171.

16. Subalakshimi, P.; Sivashanmugam, A. *ChemistrySelect* **2018**, *3*, 5040–5049.

17. Yao, X. Y.; Xin, X.; Zhang, Y. M.; Wang, J.; Liu, Z. P.; Xu, X. X. *J. Alloys Compd.* **2012**, *521*, 95–100.

18. Jadhav, H. S.; Rai, A. K.; Lee, J. Y.; Kim, J.; Park, C. J. *Electrochim. Acta* **2014**, *146*, 270–277.

19. Li, W. Y.; Xu, L. N.; Chen, J. *Adv. Funct. Mater.* **2005**, *15*, 851–857.

20. Yan, N.; Hu, L.; Li, Y.; Wang, Y.; Zhong, H.; Hu, X. Y.; Kong, X. K.; Chen, Q. W. *J. Phys. Chem. C* **2012**, *116*, 7227–7235.

21. Xu, G. L.; Li, J. T.; Huang, L.; Lin, W. F.; Sun, S. G. *Nano Energy* **2013**, *2*, 394–402.

22. Yin, X.; Wang, Z. X.; Wang, J. X.; Yan, G. C.; Xiong, X. H.; Li, X. H.; Guo, H. J. *Mater. Lett.* **2014**, *120*, 73–75.

23. Son, M. Y.; Kim, J. H.; Kang, Y. C. *Electrochim. Acta* **2014**, *116*, 44–50.

24. Lu, Z. P.; Ding, J. J.; Lin, X. H.; Liu, Y.; Ye, H. T.; Yang, G.; Yin, F.; Yan, B. *Powder Technol.* **2017**, *309*, 22–30.

25. Wang, J. Q.; Niu, B.; Du, G. D.; Zeng, R.; Chen, Z. X.; Guo, Z. P.; Dou, S. X. *Mater. Chem. Phys.* **2011**, *126*, 747–754.

26. Yang, J.; Gao, M. Z.; Lei, J. F.; Jin, X. J.; Yu, L.; Ren, F. F. *J. Solid State Chem.* **2019**, *274*, 124–133.

27. Wen, J. W.; Xu, L.; Wang, J. X.; Xiong, Y.; Ma, J. J.; Jiang, C. R.; Cao, L. H.; Li, J.; Zeng, M. *J. Power Sources* **2020**, *474*, 228491.
